# Conformity to masculine norms and symptom severity among men diagnosed with muscle dysmorphia vs. body dysmorphic disorder

**DOI:** 10.1371/journal.pone.0237651

**Published:** 2020-08-20

**Authors:** Aaron J. Blashill, William Grunewald, Angela Fang, Eliza Davidson, Sabine Wilhelm

**Affiliations:** 1 San Diego State University, San Diego, CA, United States of America; 2 San Diego State University/University of California San Diego Joint Doctoral Program in Clinical Psychology, San Diego, CA, United States of America; 3 Massachusetts General Hospital, Boston, MA, United States of America; 4 Harvard Medical School, Cambridge, MA, United States of America; Medical University of Vienna, AUSTRIA

## Abstract

Body dysmorphic disorder (BDD) is associated with severe comorbidity and impairment. Muscle dysmorphia (MD) is a subtype of BDD which has rarely been assessed outside of undergraduate student samples. Further, there are limited data comparing MD to other psychiatric disorders, including BDD. Thus, the aim of the current study is to explore differences in symptom severity and conformity to masculine norms in men diagnosed with BDD or MD. Men from the greater Boston, Massachusetts area completed a one-time assessment, which included clinician-based structured interviews and self-report questionnaires assessing MD symptom severity, BDD symptom severity, and conformity to traditional masculine norms. The sample was N = 30 men (MD: n = 15; BDD: n = 15). Statistically significant medium to large effects emerged with the MD group experiencing greater MD and BDD symptom severity, and positive attitudes towards the use of violence to solve problems. Although not reaching statistical significance, additional medium-to-large effects also emerged with the MD group reporting greater emotional restriction/suppression, heterosexual self-presentation, and desired sexual promiscuity compared to the BDD group. Findings suggest that men diagnosed with MD may experience greater MD/BDD symptom severity and endorsement of some components of ‘traditional’ masculine norms, compared to men diagnosed with BDD. Results may suggest that addressing some forms of rigid masculine norms (e.g., use of violence) in therapy could be useful in treating MD; however, additional research comparing clinical samples of men with MD and BDD are needed to guide the nosology, assessment, and treatment of MD.

## Introduction

Body dysmorphic disorder (BDD) is defined as excessive preoccupation with a perceived (or slight) defect in one’s appearance that causes significant distress and/or impairment [1]. Symptoms include repetitive behaviors, in which individuals habitually check their appearance in mirrors, assess their physical shape, make social comparisons, and/or seek reassurance due to their appearance concerns. The overall prevalence of current BDD in nationally representative samples is 1.7–2.4%, with 1.4–2.2% among men [2–4]. BDD is also associated with a host of psychiatric morbidity, including major depressive disorder, anxiety, and substance use disorders [5]. Moreover, the prevalence of suicidal thoughts and attempts are some of the highest noted across psychiatric disorders [6, 7]. The confluence of these negative health outcomes underscores the importance of further understanding the symptomatology and subtypes of BDD in order to refine prevention and intervention approaches to address this impairing disorder.

One BDD subtype is muscle dysmorphia (MD). There are limited data on the prevalence of MD; however, one study found 22% of men diagnosed with BDD met criteria for MD [9]. MD is described as a preoccupation with the thought that one is not muscular enough, coupled with a pervasive fear of muscle loss [8]. This preoccupation continues even if one is objectively muscular and may lead to withdrawal from social relationships, excessive exercise, disordered eating, and psychological distress [8]. MD is also associated with an increased risk of anabolic steroid abuse alongside polysubstance abuse, more broadly [9]. MD is currently classified as a subtype of BDD in the DSM 5 [1]; however, debate continues regarding its nosology [10–12]. This debate is due largely to most of the research on MD being conducted with non-clinical undergraduate students with self-report measures [13]. The disproportionate focus on undergraduate samples and reliance of self-report measures leaves gaps in the literature, particularly given that self-report measures alone cannot confer clinical diagnoses. Additionally, relying on a literature base which largely consists of undergraduate participants potentially limits the ability to generalize findings to a broader and diverse population (e.g., men outside the age range of 18–22 years and/or those who are not college graduates). Thus, additional research is needed with clinical samples which employ clinician-administered interviews to more accurately define BDD and MD groups.

One such study with clinical samples compared men diagnosed with BDD or MD [9]. Both groups evidenced similar levels of BDD symptom severity and delusionality; however, the MD group displayed more severe comorbid psychopathology than those with BDD. The MD group had a greater number of suicide attempts, substance abuse, and anabolic steroid misuse across their lifetimes, than the BDD group. One explanation for the greater psychopathology noted among men diagnosed with MD vs. BDD, is that men living with MD experience time-consuming and impairing behaviors characteristic of BDD (e.g., mirror-checking, reassurance seeking) in addition to behaviors that are unique to MD (e.g., excessive weightlifting, dieting to increase muscle mass, anabolic steroid misuse). It is also possible that the phenomenology of MD, compared to BDD, is more centrally connected to core aspects of male identity, and thus, preoccupation with perceived lack of muscularity may be more impairing for men than preoccupation with other aspects of appearance (e.g., skin, hair, face).

A sociocultural construct that may be important among men diagnosed with MD is adherence to traditional masculinity. For example, meta-analytic data revealed a positive and significant association between adherence to traditional masculine norms and levels of muscle dissatisfaction among men [14]. This finding suggests that men who internalize traditional norms for how men ‘should’ behave may hyper-focus on their own muscularity, given that a physique high in muscularity is often viewed as more masculine. Thus, men with high appearance orientation, who also adhere to traditional masculine norms, may focus on their muscularity as opposed to other aspects of their appearance, as an external manifestation of their masculinity. However, no known studies have compared men diagnosed with BDD to men diagnosed with MD on adherence to traditional aspects of masculinity, which may confer important information regarding treatment adaptation for MD.

There is nascent research comparing individuals diagnosed with BDD to those with MD. Indeed, there is only one known previous study [9] that makes a direct comparison between these two groups. This is a critical gap in the literature, as if research concludes that MD is associated with greater symptom severity compared to BDD, it would potentially add salience for clinicians and researchers to more thoroughly assess for the presence of MD in their patients/research studies. Additionally, if the phenomenology of MD is indeed varied from that of BDD, it may also suggest that alterations to treatment may be needed. For instance, adherence to some traditional masculine norms may be an important aspect of MD compared to BDD, which has implications for addressing schemas of masculinity in treatment of MD, which may not be necessary in cases of BDD. Thus, the current study expands on the scant literature base by contrasting BDD and MD symptom severity, and conformity to traditional masculine norms between a clinical sample of men diagnosed with BDD or MD. It is hypothesized that men with MD would score higher than men with BDD on MD symptoms and adherence to traditional masculine norms.

## Methods

### Participants and procedures

Participants were 30 adult men with a primary diagnosis of BDD (n = 15) or MD (n = 15), as determined through structured clinical assessment, who were recruited for a one-time, in-person session at Massachusetts General Hospital. Participants were recruited through flyers posted in the community (e.g., at coffee shops, bars, gyms), in addition to postings made to the hospital’s website. Prospective participants completed a phone screen to determine eligibility. On the phone screen, participants were required to be 18 years or older, speak English, and report significant preoccupation with an aspect of his appearance. After the phone screen, potential participants were scheduled for the in-person assessment, to more comprehensively determine eligibility (e.g., structured clinical assessments were administered to determine BDD/MD diagnoses). During the assessment, computerized self-report measures and clinician-based interviews were administered. Participants who met DSM-IV criteria for BDD and did not report muscularity concerns were coded in the ‘BDD group’ whereas participants who met criteria for BDD with muscularity as their primary area of concern were coded in the ‘MD group.’ All participants provided written informed consent and study procedures were approved by the Institutional Review Board of the Partners Human Research Committee (IRB # 2011-P-001692). Participants received $50 for completion of the study.

### Materials

#### Psychiatric diagnoses

The Structured Clinical Interview for DSM-IV (SCID) was administered by trained clinical interviewers in order to assess DSM-IV-based psychopathology [15], given that this study began before DSM 5 was published. The SCID for the DSM-IV has shown to be an effective tool for the diagnosis of psychiatric disorders, as well as displaying sufficient levels of categorical and dimensional inter-rater reliability [16], including BDD [17].

#### BDD symptoms

Clinically trained interviewers administered the Body Dysmorphic Disorder Modification of the Yale Brown Obsessive-Compulsive Scale (BDD-YBOCS) [18]. This scale consists of 12 items that assess BDD symptoms over the past week (e.g., preoccupation, interference, and distress regarding thoughts and behaviors about appearance concerns). Items were scored from 0–4, with higher scores representing higher levels of BDD symptoms. Phillips et al. [18] demonstrated the internal reliability, and convergent validity of this measure. Internal consistency for the BDD-YBOCS was *α* = .73.

#### MD symptoms

The Muscle Dysmorphic Disorder Inventory (MDDI) [19] is a self-report questionnaire containing 13 items that measure body image disturbance related to a perceived lack of muscle size/leanness (e.g., “I feel anxious when I miss one or more workout days”; “I wear loose clothing so that people cannot see my body”; “I think my body is too small”). Responses for the MDDI use a 5-point Likert scale ranging from “never” to “always”, with higher scores representing higher levels of MD symptoms. The MDDI shows strong test-retest reliability, divergent validity, and convergent validity [19]. Internal consistency for the MDDI was *α* = .72.

#### Traditional masculinity

The Conformity to Masculine Norms Inventory-46 (CMNI-46) [20] is a 46-item self-report measure of one’s conformity to stereotypical masculine norms, which consists of 9 subscales meant to be averaged individually [21]. Responses use a 4-point scale ranging from “strongly disagree” to “strongly agree” with higher scores reflecting greater conformity to masculine norms. Sample items include “I would feel good if I had many sexual partners”; “I frequently put myself in risky situations”; and “Sometimes violent action is necessary.” Convergent and discriminant validity as well as internal reliability of the subscales have been established [21]. The 9 subscales consist of Emotional Control, Winning, Primacy of Work, Violence, Risk-taking, Heterosexual Self-presentation, Playboy, Self-reliance, and Power Over Women. Cronbach’s alpha for these subscales ranged from *α* = .80 to *α* = .94.

#### Appearance and performance enhancing drug use

Items from the Appearance and Performance Enhancing Drug Use Schedule were administered through semi-structured interviews led by trained professionals to assess appearance and performance enhancing drug use (APED) [22]. Hildebrandt et al. found strong evidence for the internal consistency, content validity and test-retest reliability of this measure in clinical settings [22].

#### Sociodemographics

Demographic information, such as age, sexual orientation, race, ethnicity, relationship status, BMI (via objectively assessed height and weight), and level of education were collected through self-report questions.

### Statistical analyses

Models adjusted for age (given statistically significant group differences) were tested using generalized linear models with robust standard errors. In these models, BDD vs. MD was set as the independent variable and Cohen’s *d* was calculated as a metric of effect size. Cohen’s *d* is often interpreted as 0.20 = small, 0.50 = medium, and 0.80 = large. Outliers were assessed and no values were found ±3 SD from the mean. Further, normality of all outcomes was assessed and all displayed non-significant skew. An alpha level of *α* = .05 was used as the cutoff for statistical significance; however, given the small sample, we also considered effect size estimates that were medium or large to be of potential clinical importance regardless of statistical significance. Finally, given the exploratory nature of the research and paucity of preexisting literature on the topic, family-wise error was not corrected.

## Results

Regarding missing data, one participant had a missing value on one item; given this minimal level of missingness, available item analysis was employed [23]. The sample was primarily White (70%), heterosexual (63%), had some college education (83%), and had a mean age of 29.8 years (*SD* = 12.1). Sociodemographic variables and psychiatric comorbidity among the sample is reported in Table 1. Sexual minority status was over-represented in the current study (36.7% of participants), compared to the general US population, while APED use was low (MD = 13.3%, BDD = 0%). Current and lifetime substance use disorders were found among roughly 25% to 50% of the overall sample. Additionally, current and lifetime mood disorders were reported among 33.3% to 80% of participants, while current and lifetime anxiety disorders were reported among 66.7% to 70% of participants. Groups significantly differed on age *t*(28) = 2.07, *p* = .05, Cohen’s *d* = 0.76, with the BDD group older than the MD group.

**Table 1:** Demographic breakdown, and psychiatric comorbidity between participants diagnosed with muscle dysmorphia vs. body dysmorphic disorder.

Variable	MD (n = 15)	BDD (n = 15)
Age*	25.47 (5.31)	34.13 (15.32)
BMI	25.40 (2.75)	23.67 (4.38)
Sexual minority	3 (20.0%)	8 (53.3%)
White	10 (66.7%)	11 (73.3%)
Single	12 (80.0%)	13 (86.7%)
Some college+	13 (86.7%)	12 (80.0%)
APED use	2 (13.3%)	0 (0.0%)
Mood Disorder current	6 (40.0%)	4 (26.7%)
Mood Disorder lifetime	10 (66.7%)	14 (93.3%)
Anxiety Disorder current	11 (73.3%)	9 (60.0%)
Anxiety Disorder lifetime	11 (73.3%)	10 (66.7%)
Eating Disorder current	3 (20.0%)	1 (6.7%)
Eating Disorder lifetime	4 (26.7%)	2 (13.3%)
Substance Use Disorder current	4 (26.7%)	5 (33.3%)
Substance Use Disorder lifetime	6 (40.0%)	7 (46.7%)

MD = Muscle dysmorphia; BDD = Body dysmorphic disorder; APED = Appearance and Performance Enhancing Drug; BMI = Body mass index; Some college+ = some college or higher (college grad, postgraduate degree)

* denotes significant group difference at *p* = .05.

In the adjusted models, several statistically significant differences emerged between the MD and BDD groups (see Table 2). The largest effect was evidenced on MD symptoms, with the MD group reporting substantially greater symptoms than the BDD group (Cohen’s *d* = 1.69). Related, the MD group also experienced greater BDD symptom severity than the BDD group (Cohen’s *d* = 0.69). The MD group also reported substantially greater endorsement of positive attitudes towards the use of violence (Cohen’s *d* = 1.15) compared to the BDD group. Although not statistically significant, medium to large effects also emerged with the MD group reported greater desired promiscuity (Cohen’s *d* = 0.71), emotional control (Cohen’s *d* = 0.61), and heterosexual presentation (Cohen’s *d* = 0.50), compared to the BDD group.

**Table 2:** Age adjusted psychosocial comparisons between participants diagnosed with muscle dysmorphia vs. body dysmorphic disorder.

Variable	MD (n = 15)	BDD (n = 15)	Test statistic	*p* value	Effect size
Winning	1.83 (0.62)	1.65 (0.54)	*χ*^2^(1) = 0.64	.42	*d =* 0.31
Emotional Control	1.57 (0.76)	1.17 (0.53)	*χ*^2^(1) = 2.86	.09	*d =* 0.61
Primacy of Work	1.24 (0.62)	1.34 (0.81)	*χ*^2^(1) = 0.13	.72	*d = -*0.14
Risk Taking	1.47 (0.74)	1.28 (0.47)	*χ*^2^(1) = 0.61	.44	*d =* 0.31
Violence	1.87 (0.50)	1.22 (0.62)	*χ*^2^(1) = 8.51	.004	*d =* 1.15
Heterosexual Presentation	1.43 (0.82)	1.04 (0.74)	*χ*^2^(1) = 1.72	.19	*d =* 0.50
Playboy	2.02 (0.54)	1.61 (0.62)	*χ*^2^(1) = 3.30	.07	*d =* 0.71
Self-reliance	1.43 (0.74)	1.42 (0.58)	*χ*^2^(1) = 0.001	.97	*d =* 0.02
Power over Women	0.88 (0.50)	0.76 (0.70)	*χ*^2^(1) = 0.25	.61	*d =* 0.20
MDDI Total	45.60 (6.50)	34.59 (6.50)	*χ*^2^(1) = 18.95	.0001	*d =* 1.69
BDDYBOCS	27.24 (5.53)	23.69 (4.68)	*χ*^2^(1) = 3.71	.05	*d =* 0.69

*d* = Cohen’s *d*; MD = Muscle dysmorphia; BDD = Body dysmorphic disorder; BDDYBOCS = Body Dysmorphic Disorder Modification of the Yale Brown Obsessive-Compulsive Scale; MDDI = Muscle Dysmorphic Disorder Inventory

## Discussion

The current study is one of the first to compare men diagnosed with MD and BDD on symptom severity and measures of conformity to masculine norms. The majority of existing literature on MD has been based on undergraduate samples and has failed to employ clinician-based diagnostic assessments, which limits the fields’ understanding of this disorder. The MD group reported greater adherence to some aspects of traditional masculinity, including positive attitudes towards employing violence to solve problems, permissive attitudes toward multiple and frequent sexual partners, emotional restriction/suppression, and presenting oneself as heterosexual. These findings support past work that found a significant association between permissive attitudes towards multiple/frequent sexual partners, employing violence to solve problems, and muscle dissatisfaction in a sample of American men [24]. Other research has shown that men with MD reported significantly higher use of violence to solve problems and heterosexual self-presentation than men diagnosed with anorexia nervosa or a control group of gym-goers (without MD or eating disorders), and also reported significantly higher permissive attitudes toward multiple/frequent sexual partners than men with anorexia nervosa [25]. More generally, there is a positive association between greater adherence to traditional forms of masculinity and muscularity dissatisfaction [14, 26]. It may be that endorsement of violence, desire for more sexual partners, emotional restriction/suppression, and presenting one’s self as heterosexual, are salient for men diagnosed with MD. Specifically, men with MD may desire enhanced muscularity as a means to potentially enact greater violence in order to solve their problems. In addition, men with MD may desire enhanced muscularity due to a belief that sexual partners prefer a hypermuscular build, thus maximizing their chance for frequent sexual promiscuity. Related, achieving a highly muscular physique may also be a strategy to convey an outward portrayal of a ‘traditional’ masculine and hence, heterosexual, presentation. It may also be that there were 8 compared to 3 sexual minority participants in the BDD vs. MD groups; thus, the BDD group, with a higher proportion of gay and bisexual men, would likely score lower on a marker of heterosexual self-presentation compared to a group with a lower proportion of gay and bisexual men. Further, emotional restriction/suppression may be elevated among men with MD, as perhaps they utilize excessive weightlifting as a strategy to regulate negative affect. In sum, it may be that MD develops, and/or is maintained, in part, through rigid adherence to specific components of traditional masculinity, and the behavioral manifestation of MD (e.g., excessive weightlifting, use of APEDs) functions to present an outward projection of hypermasculinity.

Given the results of the current study, in concert with past research, theoretical models of MD may require modifications. Many models of MD adapt existing theories of eating disorder development, including biopsychosocial models, objectification theory, the transdiagnostic model, and the tripartite theory [27–30]. Although these models account for significant variance in MD outcomes, they typically do not include components of masculinity. Consistent with the ‘masculinity hypothesis’ [14], rigid adherence to traditional forms of masculinity is associated with elevated muscle dissatisfaction and MD symptoms [25, 31], thus, future research should consider integrated models which include salient risk factors of MD (e.g., internalization of unrealistic appearance ideals, body dissatisfaction, social comparisons, self-objectification) and aspects of traditional masculinity (e.g., violence, desired promiscuity). For example, a cognitive-affective component of internalization/over-evaluation of appearance seems to cut-across leading theories of MD, although these models do not explicitly include internalization and over-evaluation of traditional masculine norms. Incorporating aspects of rigid masculine norms into cognitive-affective factors across the aforementioned theories may aid in further understanding the development and maintenance of MD.

Further, the MD group exhibited greater BDD symptom severity and MD symptoms compared to the BDD group. It is possible that men diagnosed with MD experience greater BDD symptoms due to focal area(s) of body concern. Preoccupation with low musculature may confer, and/or be a cause of, elevated BDD symptoms, including high appearance orientation and dissatisfaction. This is in contrast to men diagnosed with BDD, whose areas of appearance concern may be more discrete, and thus, easier to camouflage than men diagnosed with MD. In the only other existing study comparing these groups, Pope et al. (2005) noted a small non-significant effect with the MD group experiencing greater BDD symptom severity than the BDD group [9]. Taken together, the data suggest that men diagnosed with MD may experience somewhat elevated symptom severity than men diagnosed with BDD; however, additional research is needed to replicate findings.

Surprisingly, APED use was relatively uncommon in this sample. Murray et al. [32] reported 90% of men diagnosed with MD indicated lifetime use of APEDs, a stark contrast to the level found in the current study. In the Murray et al. study, APED use was operationally defined to include anabolic steroids, human growth hormones, laxatives, and diuretics; whereas the current study employed a narrower definition which omitted laxatives and diuretics [32]. Although APED use has previously been linked with MD, it should be noted that individuals may experience significant distress and impairment regarding preoccupation with muscle concerns while not engaging in APED use, and thus, clinicians should not preclude a MD diagnosis based on the lack of APED use. We did not test whether the MD group statistically differed from the BDD group in APED use, as the BDD group reported 0 counts, compared to 2 in the MD group (13.3%). Future research with larger samples is needed to replicate these findings, and with a more strongly powered design, small-to-medium statistically significant differences may be revealed. Additionally, future larger studies may also wish to test if APED use is marker for more severe forms of MD, and/or may constitute a subtype within the MD classification.

The current study’s findings should be interpreted with the following limitations in mind. The sampling approach was not random or population-based, thus, estimates generated in this study should not be viewed as representative of the general population of men with BDD/MD. Additionally, the sample size was modest, and there was low statistical power to detect statistically significant effects. Even the medium-to-large effects which were found should be interpreted cautiously, as with the small sample size, the precision of these estimates are likely variable. Further, although one of the strengths of the current study is the use of clinician-based structured interviews, inter-rater reliability was not conducted, and thus, error associated with the trained clinician’s assessment is not known. However, the assessor was formally trained in all clinical interviews, is a licensed psychologist, and has extensive research and experience with BDD/MD. Lastly, the design of the study was cross-sectional, and thus, inferences regarding temporal ordering of variables cannot be made. Future research would benefit from prospective designs in which traditional forms of masculinity and MD/BDD symptoms are explored over time to examine potential mechanisms among these variables.

The results from the current study may impart some clinical implications. Specifically, the divergence in traditional masculinity may warrant consideration. There are no known empirically supported treatments for MD, per se; however, cognitive behavioral therapy (CBT) for BDD has demonstrated strong empirical support for its efficacy [33]. Modular-based CBT for BDD is a promising treatment which includes a module on MD; however, to date, there is no data on whether the efficacy of CBT varies as a function of MD diagnosis [34, 35]. Findings from the current study may indicate that addressing masculinity in therapy may be a fruitful approach. Identifying patients’ schemas about what it means to ‘be a man’ and working to modify rigid, maladaptive beliefs about masculinity may have synergistic effects with addressing core BDD symptoms. However, additional research is needed with larger clinical samples to support these suggestions.

## Conclusions

The current study assessed similarities and differences between men clinically diagnosed with BDD or MD. Overall, there was some evidence to suggest that men with MD may be more traditional in masculinity (specifically endorsement of violence, desired promiscuity, emotional restriction/suppression, and heterosexual self-presentation) and may experience greater BDD symptomatology compared to men with BDD. Future research is needed with larger clinical samples, and existing frontline treatment for BDD and should be tested within MD samples.
